# Exosomal miR-92b-3p Promotes Chemoresistance of Small Cell Lung Cancer Through the PTEN/AKT Pathway

**DOI:** 10.3389/fcell.2021.661602

**Published:** 2021-05-31

**Authors:** Ming Li, Wulin Shan, Yan Hua, Fengmei Chao, Yayun Cui, Lei Lv, Xiaoyan Dou, Xing Bian, Jinglu Zou, Hong Li, Wenchu Lin

**Affiliations:** ^1^Department of Laboratory Diagnostics, The First Affiliated Hospital of USTC, Division of Life Sciences and Medicine, University of Science and Technology of China, Hefei, China; ^2^High Magnetic Field Laboratory, Hefei Institutes of Physical Science, Chinese Academy of Sciences, Hefei, China; ^3^Key Laboratory of High Magnetic Field and Ion Beam Physical Biology, Hefei Institutes of Physical Science, Chinese Academy of Sciences, Hefei, China

**Keywords:** exosome, miR-92b-3p, small cell lung cancer, chemoresistance, migration

## Abstract

Resistance to first-line chemotherapy drugs has become an obstacle to improving the clinical prognosis of patients with small cell lung cancer (SCLC). Exosomal microRNAs have been shown to play pro- and anti-chemoresistant roles in various cancers, but their role in SCLC chemoresistance has never been explored. In this study, we observed that the expression of exosomal miR-92b-3p was significantly increased in patients who developed chemoresistance. Luciferase reporter analysis confirmed that PTEN was a target gene of miR-92b-3p. The PTEN/AKT regulatory network was related to miR-92b-3p-mediated cell migration and chemoresistance *in vitro* and *in vivo* in SCLC. Importantly, exosomes isolated from the conditioned medium of SBC-3 cells overexpressing miR-92b-3p could promote SCLC chemoresistance and cell migration. Furthermore, we found that plasma miR-92b-3p levels were significantly higher in patients with chemoresistant SCLC than in those with chemosensitive SCLC, but the levels were down-regulated in patients who achieved remission. Kaplan–Meier analysis showed that SCLC patients with high miR-92b-3p expression were associated with shorter progression-free survival. Overall, our results suggested that exosomal miR-92b-3p is a potential dynamic biomarker to monitor chemoresistance in SCLC and represents a promising therapeutic target for chemoresistant SCLC.

## Introduction

Lung cancer, one of the most common cancers worldwide, leads to high cancer-related death among both men and women (Siegel et al., 2019). Small cell lung cancer (SCLC) is a neuroendocrine tumor that accounts for approximately 15% of all lung cancers (Sun et al., 2017). SCLC is the most aggressive lung malignancy, exhibiting a strong proliferative capacity, early distant migration, and drug resistance, all of which lead to extremely poor outcomes for SCLC patients. The first line of treatment regimen for SCLC is platinum-based chemotherapy, usually in combination with the topoisomerase II inhibitor etoposide (William and Glisson, 2011; Borromeo et al., 2016). The treatment regimen shows a good initial response in 60–80% SCLC patients, but almost all patients relapse within 6–12 months of treatment due to the development of multidrug resistance (MDR; Peng et al., 2016). The indicators currently used for monitoring SCLC chemoresistance are imaging data and traditional tumor markers, such as carcinoembryonic antigen, pro-gastrin-releasing peptide, and neuron-specific enolase. Owing to radiation hazards, imaging cannot be performed as a routine examination during chemotherapy. Most traditional tumor markers lack high sensitivity and specificity. Therefore, it is urgent to find reliable predictors and elucidate the molecular mechanism of SCLC chemoresistance.

MicroRNAs (miRNAs) are endogenously derived non-coding RNAs with 19–22 nucleotides. Numerous studies have revealed that miRNAs are involved in cell proliferation, apoptosis, drug resistance, cancer migration, and progression (Wei et al., 2017; Liu et al., 2019). The relationship between miR-92b-3p expression and cancer development remains controversial. Some reports suggest that miR-92b-3p expression is increased in most cancers, including colorectal cancer (Gong et al., 2018), esophageal squamous cell cancer (Wang et al., 2019), gastric cancer (Li C. et al., 2019), non-SCLC (NSCLC; Lei et al., 2014), and clear cell renal cell carcinoma (Wang et al., 2020). In contrast, miR-92b-3p is confirmed as a tumor suppressor miRNA in pancreatic cancer (Long et al., 2017) and triple-negative breast cancer (Li Y. Y. et al., 2019). However, the specific function of miR-92b-3p in SCLC is not yet understood.

Exosomes ranging from 30 to 150 nm in diameter are widely present in various bodily fluids. Exosomes can be produced by tumor, epithelial, T, B, and dendritic cells. Most studies have demonstrated that the exosomes secreted by tumor cells could migrate far away from their initial position and then transfer various types of functional effectors, including miRNAs, proteins, and lipids, to recipient cells. In addition, exosomal miRNAs have been considered as the most effective biomarkers for disease diagnosis and efficacy monitoring in the past few years (Joyce et al., 2016; Mao et al., 2020). However, the relationship between tumor-derived exosomal miRNAs and chemoresistance in SCLC has not been elucidated.

In the present study, we first isolated total RNA from plasma exosomes of two SCLC patients at the pre- and post-chemoresistant stages and used next-generation sequencing (NGS) to examine the differential expression of miRNAs. We found that the levels of miR-92b-3p in plasma exosomes were significantly increased in SCLC patients with chemoresistance compared with patients without chemoresistance. Hence, we focused on exosomal miR-92b-3p and studied its effect on SCLC chemoresistance. The data showed that miR-92b-3p could be transferred among cancer cells via exosomes to promote cell migration and chemoresistance through the PTEN/AKT pathway *in vitro* and *in vivo*. Additionally, miR-92b-3p was demonstrated to be significantly up-regulated in both the plasma and plasma exosomes of SCLC patients with chemoresistance. Our study reveals novel details elaborating the molecular mechanism of exosomal miR-92b-3p in SCLC chemoresistance and offers theoretical basis for identifying effective targets to improve the prognosis of SCLC chemoresistant patients.

## Materials and Methods

### Cell Lines, Materials, and Antibodies

The human SCLC cell lines H82, SHP77, DMS273, and H69 were kept in our own laboratory. SBC-3 and H446 cells were purchased from the Cell Bank of Type Culture Collection of the Chinese Academy of Sciences (Shanghai, China). Cells were maintained in Roswell Park Memorial Institute (RPMI) 1640 medium (Life Technologies, CA, United States) supplemented with 10% fetal bovine serum (FBS; Gibco, Australia) and 1% penicillin/streptomycin in a humidified incubator at 37^°^C in 5% CO_2_.

Cisplatin (DDP), adriamycin (ADM), and etoposide (VP16) were purchased from MCE (Concord, CA, United States), and stock solutions were prepared in dimethyl sulfoxide (Sigma-Aldrich, Saint Louis, Mo, United States) at a concentration of 1 mg/ml. Antibodies against CD63, TSG101, PTEN, phospho-AKT, and AKT were purchased from Cell Signaling Technology (Danvers, MA, United States). GAPDH antibody was purchased from TransBionovo (Beijing, China). The bicinchoninic acid (BCA) protein assay was purchased from Thermo Fisher Scientific (CA, United States).

### MiR-92b-3p Transfection

SBC-3 and SHP77 cells were seeded 1 day before transfection. Lentivirus vectors containing the miR-92b-3p mimic (Lv3-miRNA-92b-3p mimics), miR-92b-3p inhibitor (Lv3-miRNA-92b-3p inhibitor sponge), or NC (Lv-NC) were amplified by GenePharma (Shanghai, China). All these plasmids and oligonucleotides were transfected into cells by Lipofectamine 3000 reagent (Invitrogen) following the manufacturer’s instructions, and cells were continually incubated with puromycin (2.5 μg/ml, Sigma) to develop acquired resistance.

### Western Blot and Immunohistochemistry Assays

For the western blot assay, total proteins were extracted using extraction buffer with a protease inhibitor cocktail (Thermo Scientific), and their concentration was quantified with the BCA assay. Protein of 50 μg was separated by sodium dodecyl sulfate (SDS)–polyacrylamide gel electrophoresis and then transferred to polyvinylidene fluoride membranes (Millipore). The membranes were blocked and probed overnight with primary antibodies, including CD63, TSG101, PTEN, and AKT (T/P). After incubation with secondary antibodies, the protein band intensity was quantified by densitometry using Image Lab software (Bio-Rad, Hercules, CA, United States).

For the immunohistochemistry (IHC) assay, tissue blocks were sectioned at a thickness of 4 μm, and the sections were deparaffinized. Briefly, the sections were incubated in xylene, followed by ethanol, and then washed with distilled water. For antigen retrieval, the sections were boiled in 10 mM of sodium citrate buffer for 10 min at 121^°^C. After being rinsed with distilled water, the sections were incubated in 3% peroxidase before they were washed with distilled water followed by buffer. For staining, sections were exposed to primary antibody targeting PTEN or p-AKT (1:800) diluted in an antibody diluent. For antigen visualization, a peroxidase-labeled secondary antibody (EnVision/HRP system, DAKO, Carpinteria, CA, United States) was applied. Subsequently, the sections were rinsed in the buffer provided in the kit and immersed in a 3,3′-diaminobenzidine staining solution.

### Quantitative Real-Time Polymerase Chain Reaction

Total RNA was isolated from cells using TRIzol. RNA from plasma or exosomes was isolated using the miRNeasy Micro Kit (Qiagen). cDNA was synthesized with the TaqMan^®^ MiRNA Reverse Transcription Kit (Applied Biosystems). Aliquots of the reaction mixture were used for PCR on an LC480 PCR Detection System. All primers were synthesized by RiboBio (Guangzhou, China). All PCR experiments were performed in triplicate. The U6 RNA levels for cellular miR-92b-3p, miR-39 levels for plasma, and exosomal miR-92b-3p and GAPDH levels for mRNAs were used as respective internal controls for data normalization.

### Exosome Purification and Identification

The isolation of SCLC supernatant exosomes was adapted from a previous study (Lobb et al., 2015). For exosome purification from cell culture supernatants, cells were cultured in medium containing 10% exosome-depleted FBS. In brief, culture supernatants were centrifuged at 300 × *g* for 10 min to remove living cells and 2,000 × *g* for 20 min to remove cell debris and dead cells. Microvesicles were pelleted after centrifugation at 16,500 × *g* for 45 min at 4^°^C (Beckman Coulter, J2-HS) and resuspended in phosphate-buffered saline (PBS). Supernatants filtered through a 0.22-μm membrane were then centrifuged at 100,000 × *g* for 2 h at 4^°^C (Beckman Coulter, Optima XPN-100). The pelleted exosomes were resuspended in PBS and collected by ultracentrifugation at 100,000 × *g* for 2 h. To isolate exosomes from the peripheral blood, samples were centrifuged at 2,000 × *g* for 10 min two times to separate the plasma, and exosomes were isolated via ultracentrifugation as described above.

Protein levels in exosomes were quantified by the BCA assay, and western blot analysis of exosome-related proteins CD63 and TSG101 was performed following standard procedures. Exosomes suspended in sucrose gradients were prepared as described above and resuspended in PBS. Sample aliquots of 4 ml were pipetted onto 200 mesh formvar/carbon grids (EMS), which had been subjected to glow discharge for 15 s. Samples were incubated on grids for 30 s and subsequently negatively stained with a 2% uranyl acetate solution. Data were acquired using a Philips CM200F electron microscope operating at 200 keV equipped with an UltraScan 1000 CCD camera (Gatan). Exosomes were quantified by a NanosightNS300 instrument (Malvern Instruments Ltd., United Kingdom) equipped with NTA 3.0 analytical software (Malvern Instruments Ltd., United Kingdom).

### Cell Viability, Apoptosis, Migration, and Invasion Assays

For the cell viability assay, SCLC cells (1 × 10^4^) were cultured in 96-well plates under the indicated experimental conditions. Cells were incubated with different concentrations of chemotherapy drugs, including DDP, ADM, and VP16. After 24 h, Cell Counting Kit 8 (CCK8, Dojindo, Japan) was used to detect cell viability. Then, the IC50 of each drug was calculated.

For the apoptosis assay, SCLC cells were treated with 3 μg/ml of DDP for 48 h and then collected. Annexin V/7-AAD (eBioscience, United States) was used according to the manufacturer’s protocol.

Wound-healing analysis was performed to test cell migration. The artificial wounds were produced on aconfluent cell monolayer in FBS-free medium. Cell invasion was assessed using Transwell chambers according to the manufacturer’s instructions. Briefly, SCLC cells (2 × 10^5^ cells/well) were seeded in the upper chambers on a Matrigel-coated membrane in FBS-free medium. Meanwhile, the lower chambers were loaded with RPMI 1640 medium supplemented with 10% FBS. After incubation at 37^°^C for 48 h, cells remaining on the upper surface of the membrane were cleaned with a cotton swab. The lower chamber was washed with PBS, fixed with methyl alcohol, stained with 0.5% crystal violet, washed three times with water, and viewed under an inverted microscope.

### Luciferase Reporter Assay

HEK 293T cells were co-transfected with miR-92b-3p mimics or with a non-specific control (NC; GenePharma) and wild-type or mutated PTEN 3′-untranslated region (3′-UTR) plasmids (constructed by GenePharma) using Lipofectamine 3000 (Invitrogen, United States). Luciferase activities were measured at 48 h post-transfection using a dual-luciferase analysis system (Promega, Madison, WI, United States). Luminescence readings were acquired using a FlexStation 3 Multiscan Spectrum (Molecular Devices, Sunnyvale, CA, United States).

### Co-cultured Assays

To investigate the role of AKT, SCLC cells were cultured in 6-well or 96-well plates treated with the p-AKT inhibitor MK-2206 alone or in combination with DDP. After 48 h, cells were harvested and subjected to cell viability, migration, qRT-PCR, and western blot assays.

To investigate the potential transmission of chemoresistance, SCLC cells were seeded in 6-well plates and incubated with exosomes isolated from the culture supernatant of SCLC with miR-92b-3p overexpression (SCLC OE) cells in RPMI 1640 medium with 10% exosome-depleted FBS. Then, the cells were harvested for subsequent wound healing assays and measurement of miR-92b-3p and PTEN levels.

### Exosome Uptake Assay

Exosomes were labeled with the red fluorescent dye PKH26 (Sigma-Aldrich) according to the manufacturer’s protocol. Briefly, exosomes isolated from the culture supernatant of SBC-3 OE cells were resuspended in 0.5 ml of Diluent C. Then, 2 μl of PKH26 was diluted in another 0.5 ml of Diluent C. The samples were mixed for 5 min, and then 5 ml of 1% bovine serum albumin was added to quench excess dye. Subsequently, the mixture was ultracentrifuged at 100,000 × *g* for 1 h, resuspended in PBS, and finally incubated with sensitive cells for 12 h at 37^°^C. Incorporation of exosomes into cells treated with DAPI was visualized by fluorescence microscopy (Carl Zeiss, Germany).

### Animal Experiments

Animal experiments were approved by the Institutional Animal Care and Use Committees of Hefei Institutes of Physical Science, Chinese Academy of Sciences. 4-week-old female BALB/c nude mice were purchased from Beijing Vitong Lihua Laboratory Animal Technology Co., Ltd (Beijing, China).

To evaluate whether miR-92b-3p could promote SCLC tumor growth, SBC-3 NC, SBC-3 OE, and SBC-3 with miR-92b-3p knockdown (SBC-3 KD) cells (5 × 10^6^ cells per point) were subcutaneously injected into the right upper and lower flanks of nude mice. Tumor growth was monitored two times per week. After 4 weeks, mice from each group were sacrificed, and their tumor samples were prepared for histological examination.

To evaluate whether exosomes isolated from the culture supernatant of SBC-3 OE cells could promote SCLC chemoresistance, SBC-3 cells (5 × 10^6^ cells per mouse) were injected subcutaneously into the right upper and lower flanks of nude mice. 10 days later, when the tumors were approximately 100 mm^3^ in size, purified exosomes (5 μg) or PBS was then injected intratumorally twice weekly with or without DDP (3.5 mg/kg). The tumor size was measured twice per week. Tumor volume (mm^3^) was calculated as 0.5 × width^2^ × length.

### Clinical Small Cell Lung Cancer Patient Samples

Fifty SCLC patients were enrolled in the Department of Respiratory Oncology of the Western Branch of the First Affiliated Hospital of University of Science and Technology of China (Hefei, China) from January 2016 to July 2019. The clinical and pathological characteristics of these patients are listed in Table 1. All patients received routine platinum agents combined with VP16 chemotherapy. The treatment response was divided into responder and non-responder groups. The responder group included patients who achieved a partial response (PR) or complete response (CR), and the non-responder group included patients with stable disease (SD) and progressive disease (PD). This study was approved by the Ethics Committee of the First Affiliated Hospital of University of Science and Technology of China.

**TABLE 1:** Univariate and multivariate prognostic analyses of SCLC.

**Variables**	**Univariate analysis HR (95% CI)**	***P* value**	**Multivariate analysis HR (95% CI)**	***P* value**
Sex male vs female	1.144 (0.621–2.108)	0.666		
Age < 64 vs ≥64	1.003 (0.981–1.026)	0.797		
Smoking yes vs no	0.759 (0.455–1.266)	0.291		
Stage LD vs ED	2.029 (1.108–3.716)	0.022	1.737 (0.928–3.251)	0.084
MiR-92b-3p high vs low	0.517 (0.341–0.851)	0.010	0.590 (0.353–0.988)	0.045

*Note. SCLC, small cell lung cancer; LD, limited disease; ED, extensive disease.*

### Statistical Analysis

All statistical analyses were performed using GraphPad Prism software 8.5 (San Diego, CA, United States) and SPSS 21.0 (IBM Corporation, Armonk, NY, United States). The Mann–Whitney *U* test was performed to compare the levels of plasma and exosomal miR-92b-3p. The clinicopathological parameters were compared using Fisher’s exact test. The statistical significance between two groups was analyzed using two-tailed unpaired Student’s t test. Survival curves were constructed with the Kaplan–Meier method and compared by log-rank tests. *P* < 0.05 was considered statistically significant.

## Results

### MiR-92b-3p Is Aberrantly Up-Regulated in the Plasma Exosomes of Small Cell Lung Cancer Patients With Chemoresistance

**FIGURE 1:**
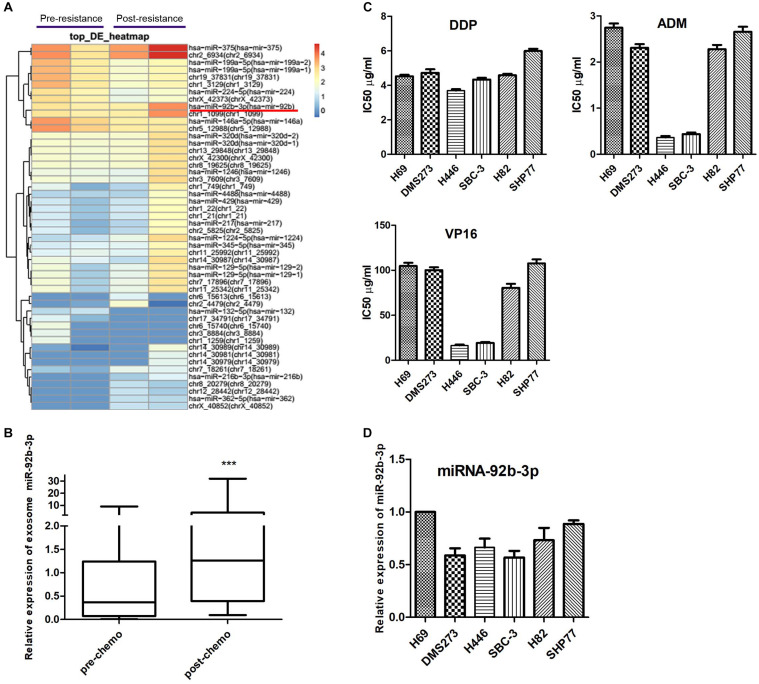
Circulating miR-92b-3p is aberrantly up-regulated in SCLC chemoresistant patients. **(A)** The heatmap shows the top miRNAs with the largest changes (increased or decreased) in expression in plasma exosomes of two patients before and after developing chemoresistance. **(B)** The level of miR-92b-3p was higher in plasma exosomes from chemoresistant patients than from chemosensitive patients. ***P < 0.001. **(C)** IC50 values of DDP, ADM, and VP16 in SCLC cell lines. SBC-3 and H446 cells were relatively sensitive to chemotherapy drugs, while SHP77 cells were resistant to chemotherapy drugs. **(D)** MiR-92b-3p expression in SCLC cell lines. MiR-92b-3p expression was higher in chemoresistant SHP77 cells than in the other cell lines, whereas the lowest expression was observed in chemosensitive SBC-3 cells. **(B–D)** experiments were performed independently three times. SCLC, small cell lung cancer; miRNAs, microRNAs; DDP, cisplatin; ADM, adriamycin; and VP16, etoposide.

Total RNA was isolated from plasma exosomes of two patients at the pre- and post-chemoresistant stages. MiRNA sequencing was performed to assess differential expression using NGS. The data showed that the levels of plasma exosomal miR-92b-3p were much higher in the post-chemoresistant stage than in the pre-chemoresistant stage (Figure 1A). To further explore the level of circulating miR-92b-3p in SCLC patients, another 20 plasma samples from SCLC patients at the pre- and post-chemoresistant stages was collected for exosome isolation. Consistent with the sequencing data, plasma exosomal miR-92b-3p levels were aberrantly increased after patients developed chemoresistance (Figure 1B).

To identify SCLC cell lines with differential responses to first-line chemotherapy drugs, a panel of six cell lines was screened using the CCK8 assay. As shown in Figure 1C, there was no significant difference in the IC50 of DDP among the SCLC cell lines, whereas the IC50 values of ADM and VP16 in SBC-3 and H446 cells were significantly lower than those of other cell lines, especially of SHP77 cells. The data indicated that SBC-3 and H446 cells were relatively sensitive to chemotherapy drugs, while SHP77 cells were resistant to these drugs. We further investigated miR-92b-3p expression in these six cell lines. Consistent with the data in plasma exosomes, miR-92b-3p expression was higher in chemoresistant SHP77 cells, whereas the lowest expression was observed in chemosensitive SBC-3 cells (Figure 1D). Our data implied the potential importance of miR-92b-3p in promoting SCLC chemoresistance.

### MiR-92b-3p Is Involved in Chemoresistance Regulation in Small Cell Lung Cancer Cells

**FIGURE 2:**
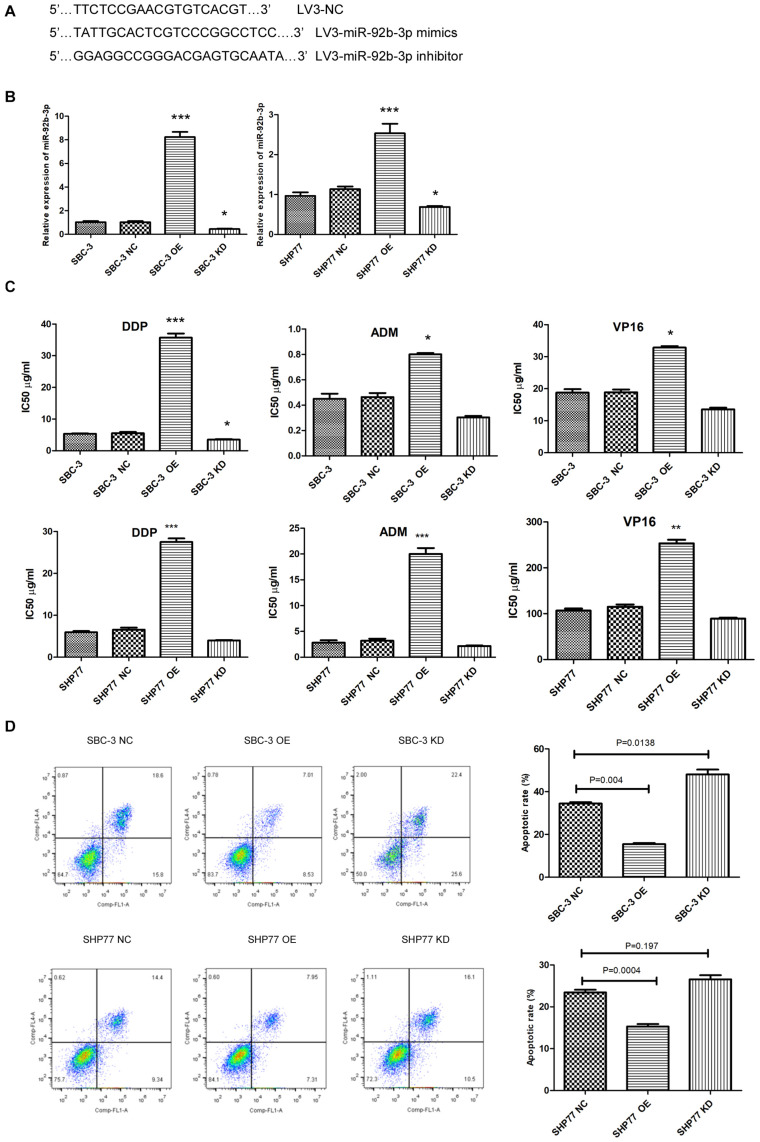
MiR-92b-3p promotes chemoresistance and inhibits apoptosis in SCLC cells. **(A)** Primer sequences of lentiviral miR-92b-3p. **(B)** The miR-92b-3p levels were increased in SCLC OE cells and decreased in SCLC KD cells. **(C)** IC50 of DDP, ADM, and VP16 in SCLC cells. MiR-92b-3p promoted chemoresistance in SCLC cells. **(D)** MiR-92b-3p inhibited SCLC cell apoptosis. OE, miR-92b-3p overexpression; KD, miR-92b-3p knockdown. **(B–D)** analyses were performed in triplicate. SCLC, small cell lung cancer; DDP, cisplatin; ADM, adriamycin; and VP16, etoposide. *P < 0.05, **P < 0.01, ***P < 0.001.

**FIGURE 3:**
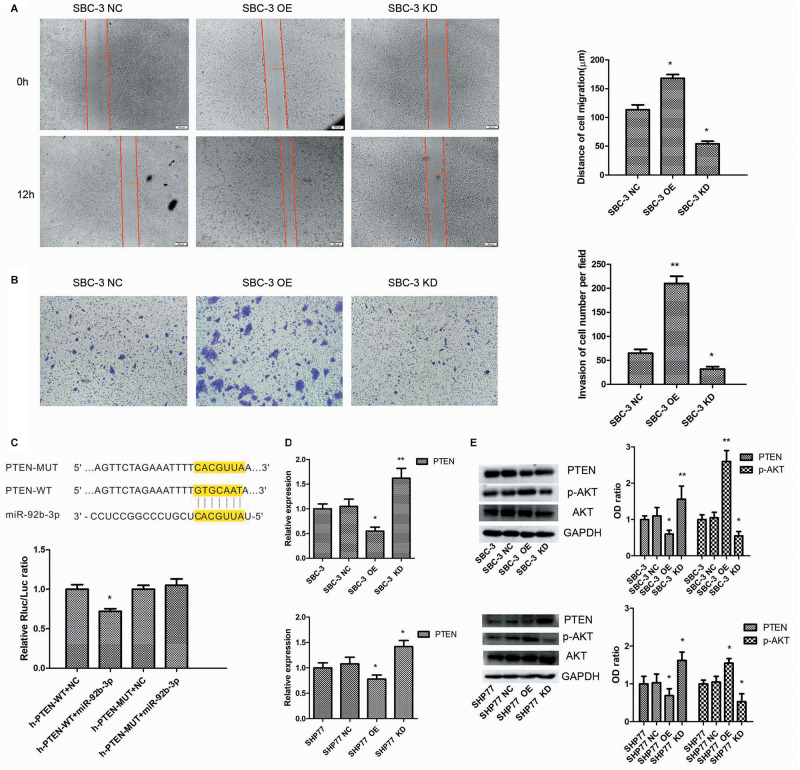
MiR-92b-3p promotes SCLC chemoresistance by targeting the PTEN/AKT pathway. **(A,B)** MiR-92b-3p promotes SBC-3 cell migration and invasion. **(C)** The highly conserved miR-92b-3p binding motif in the 3′-UTR of PTEN. MiR-92b-3p only suppressed the luciferase activity in cells expressing the wild-type 3′-UTR of PTEN. **(D)** qRT-PCR data showed that PTEN expression was increased in SCLC KD cells but decreased in SCLC OE cells. **(E)** PTEN, AKT, and p-AKT levels were detected by western blot. The p-AKT level was significantly decreased in SCLC KD cells, whereas the PTEN level was increased in SCLC OE cells. **(A,B,D,E)** data points were determined three times. SCLC, small cell lung cancer. *P < 0.05, **P < 0.01.

We first used lentivirus transfection to construct SCLC cell lines with stable overexpression or knockdown of miR-92b-3p. After transfection, SCLC cells presented green fluorescence. The mimic and inhibitor fragments of miR-92b-3p are shown in Figure 2A. The expression of miR-92b-3p in SBC-3 OE and SHP77 OE cells was 8 and 2.54 times than that in the respective NC cells. Conversely, miR-92b-3p levels in SBC-3 KD and SHP77 KD cells were 30% and 50% of that in NC cells, respectively, (Figure 2B). After miR-92b-3p overexpression, the IC50 values of DDP, ADM, and VP16 were significantly increased in SBC-3 and SHP77 cells. Only the IC50 of DDP in SBC-3 KD cells was dramatically decreased, whereas the IC50 values of all the chemotherapy drugs were not observably changed in SHP77 KD cells (Figure 2C). Moreover, after treatment with 3 μg/ml of DDP, the apoptosis rate was significantly decreased in SBC-3 OE cells and SHP77 OE cells. By contrast, the apoptosis rate was dramatically increased in SBC-3 KD cells, whereas the rate was not observably changed in SHP77 KD cells (Figure 2D). Wound healing and transwell assays demonstrated that SBC-3 KD cells showed significantly less migration and lower invasion ability, whereas the effects were reversed after miR-92b-3p overexpression (Figures 3A,B). The data shown above revealed that miR-92b-3p overexpression markedly enhanced chemoresistance in SCLC cells.

### MiR-92b-3p Promotes Small Cell Lung Cancer Chemoresistance Through the PTEN/AKT Pathway *in vitro* and *in vivo*

We next explored the molecular mechanisms by which miR-92b-3p promoted SCLC chemoresistance. Bioinformatics analysis showed that the PTEN 3′-UTR contains sites complementary to miR-92b-3p (Figure 3C). To verify this prediction, we separately cloned fragments of the PTEN 3′-UTR that contained wild-type or mutated miR-92b-3p-binding sequences into psi-CHECK2 luciferase reporter plasmids. As shown in Figure 3C, the luciferase activities were significantly inhibited by miR-92b-3p in the plasmid carrying wild-type target sequences, whereas this inhibition was abrogated when the predicted sequences were mutated. Western blot and qRT-PCR data showed that the PTEN levels were increased in SBC-3 KD and SHP77 KD cells, and the levels were observably decreased in miR-92b-3p-overexpressing cells (Figures 3D,E). Additionally, we found that the p-AKT levels were significantly decreased in SBC-3 KD and SHP77 KD cells but were increased after miR-92b-3p overexpression (Figure 3E).

**FIGURE 4:**
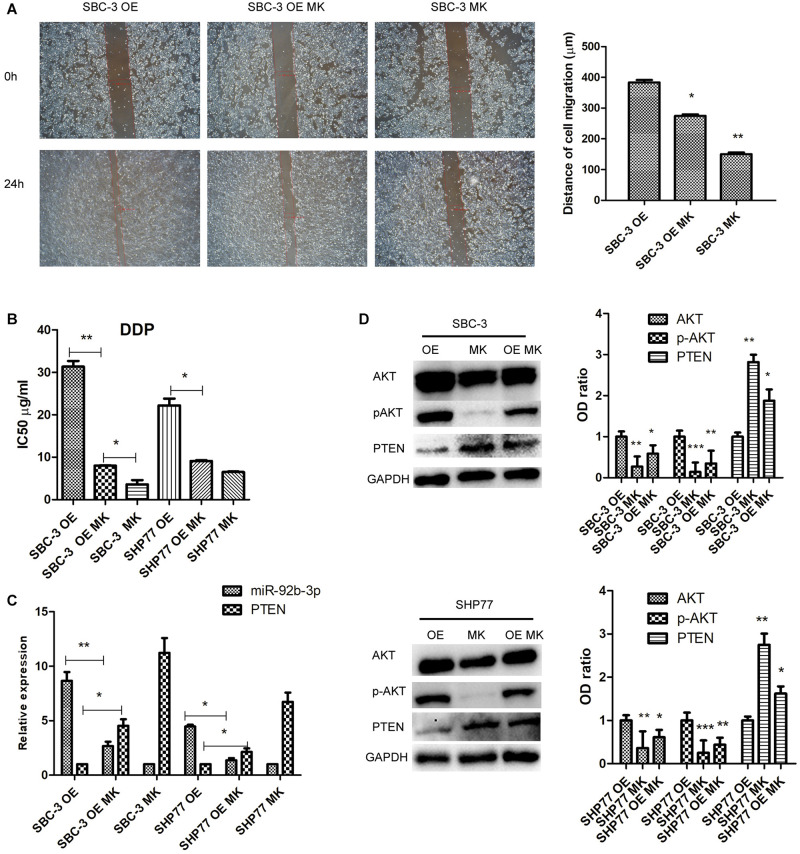
p-AKT downregulation inhibited cell migration and chemoresistance of SCLC. **(A)** p-AKT downregulation inhibited SBC-3 OE and SBC-3 cell migration. **(B)** p-AKT downregulation decreased the IC50 value of DDP in SBC-3 and SHP77 cells, whereas this decrease was attenuated in SCLC OE cells. **(C)** MiR-92b-3p and PTEN levels as detected by qRT-PCR. p-AKT downregulation inhibited miR-92b-3p expression and enhanced PTEN levels in SCLC OE cells. **(D)** AKT, p-AKT, and PTEN levels as detected by western blot. p-AKT knockdown reduced p-AKT levels and increased PTEN levels in SCLC cells, but this was reversed by miR-92b-3p overexpression. All analyses were performed in triplicate. SCLC, small cell lung cancer and DDP, cisplatin. *P < 0.05, **P < 0.01, ***P < 0.001.

To further investigate the effects of PTEN/AKT pathway on SCLC cellular function and chemoresistance, we primarily reduced p-AKT expression using the p-AKT inhibitor MK-2206 in SCLC cells. After inhibition of p-AKT activity, SBC-3 OE and SBC-3 cells showed reduced migration ability (Figure 4A). In addition, p-AKT downregulation observably decreased the IC50 values of DDP in SBC-3 and SHP77 cells, whereas this decrease was attenuated in SCLC cells overexpressing miR-92b-3p (Figure 4B). qRT-PCR data showed that p-AKT downregulation markedly inhibited miR-92b-3p expression and enhanced PTEN levels in SBC-3 OE and SHP77 OE cells (Figure 4C). p-AKT knockdown significantly reduced p-AKT levels in SBC-3 and SHP77 cells, whereas this reduction was attenuated in SBC-3 OE and SHP77 OE cells. Conversely, the PTEN levels in SCLC cells were enhanced after inhibiting p-AKT expression, whereas this increase was attenuated in SCLC OE cells (Figure 4D). These results indicated that p-AKT expression mediated cell migration, chemoresistance, and miR-92b-3p and PTEN levels in SCLC cells.

**FIGURE 5:**
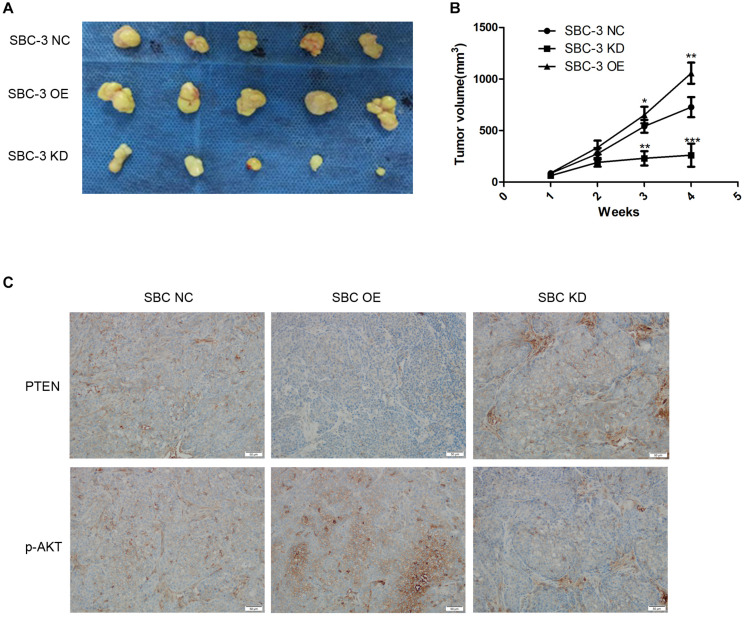
MiR-92b-3p promotes SCLC development through the PTEN/AKT pathway *in vivo*. **(A,B)** The tumor-forming ability of SBC-3 cells with different miR-92b-3p expression levels. MiR-92b-3p promoted the tumor growth *in vivo.* Compared with SBC-3 NC group, *P < 0.05, **P < 0.01, and ***P < 0.001. **(C)** After miR-92b-3p overexpression, the IHC data confirmed that PTEN levels were down-regulated while p-AKT levels were up-regulated in tumor tissue. SCLC, small cell lung cancer and IHC, immunohistochemistry.

We finally studied the function of miR-92b-3p in SCLC *in vivo*. We performed a tumorigenesis assay by subcutaneously injecting cells (SBC-3 NC, SBC-3 OE, and SBC-3 KD) into the flanks of nude mice. The results showed that tumor growth was slowed by miR-92b-3p knockdown but was accelerated by miR-92b-3p overexpression (Figures 5A,B). The expression of PTEN and p-AKT was observed in the tumor tissue samples by IHC. The data confirmed that PTEN expression was down-regulated whereas p-AKT levels were increased in the SBC-3 OE group (Figure 5C). Conversely, these proteins showed an opposite trend in the SBC-3 KD group. Based on the above results, we concluded that miR-92b-3p promoted SCLC development and chemoresistance by regulating PTEN/AKT signaling.

### MiR-92b-3p Is Transferred via Exosomes to Promote Cell Migration and Chemoresistance in Small Cell Lung Cancer

**FIGURE 6:**
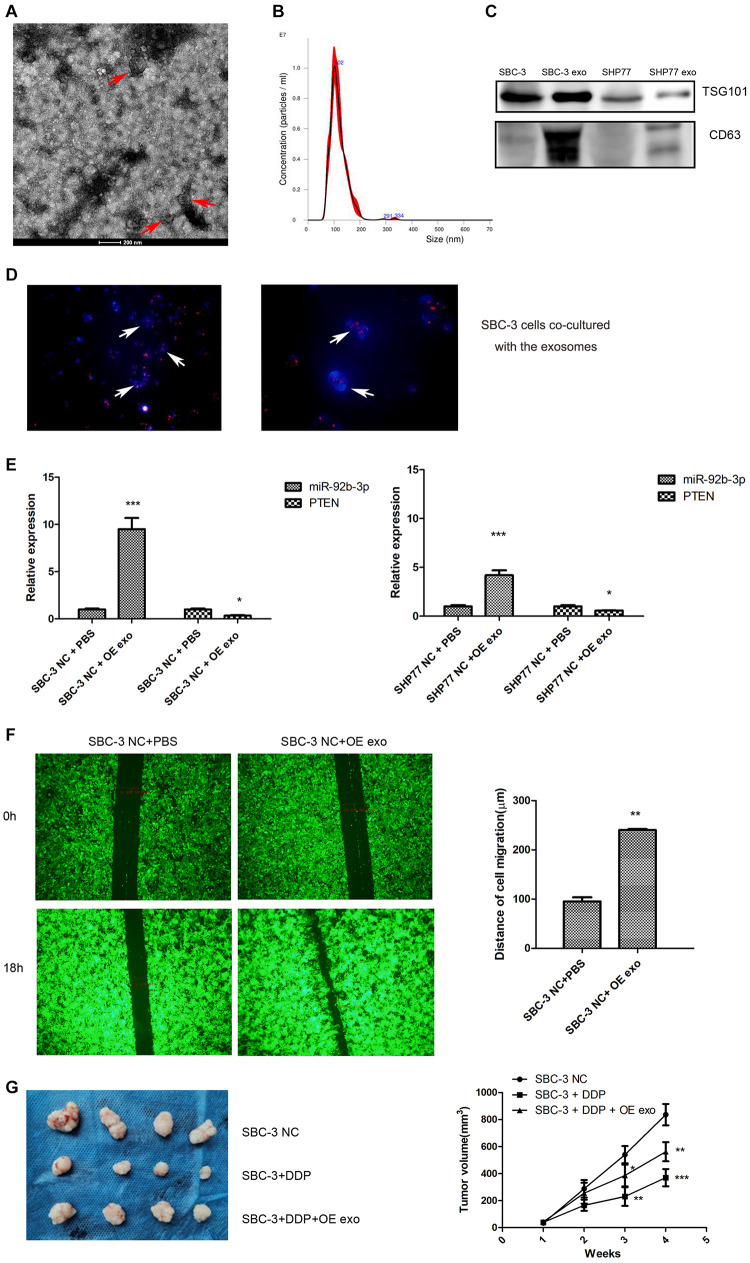
Exosomes transfer miR-92b-3p to promote SCLC migration and chemoresistance. **(A)** TEM images of exosomes isolated from SCLC cells. **(B)** The size and quantitation of exosomes isolated from SCLC cells were examined by nanoparticle tracking analysis. **(C)** The levels of CD63 and TSG101 were detected by western blot. **(D)** Fluorescence microscopy images of SBC-3 cells after treatment with PKH26-labeled exosomes (green) stained with DAPI (blue). SBC-3 cells could effectively uptake the exosomes. **(E)** MiR-92b-3p and PTEN levels as detected by qRT-PCR. SCLC cells showed higher levels of miR-92b-3p and lower levels of PTEN after they were incubated with exosomes. **(F)** SCLC cells displayed higher migration ability after incubation with exosomes. **(G)** DDP treatment inhibited tumor growth, whereas this inhibition was reversed after combination therapy with exosomes. Compared with SBC-3 NC group, *P < 0.05, **P < 0.01, and ***P < 0.001. All experiments were performed independently three times. SCLC, small cell lung cancer and TEM, transmission electron microscopy.

To determine the function of exosomal miR-92b-3p in SCLC development and chemoresistance, we conducted a series of experiments. First, exosomes from the supernatant of cultured SCLC cells were isolated. The isolated exosomes exhibited typical cup-shaped morphology and size (Figures 6A,B) and also positively expressed the protein markers CD63 and TSG101 (Figure 6C). The exosomes isolated from the conditioned medium of SBC-3 OE cells were labeled with PKH26 and co-cultured with SBC-3 cells for 24 h. Immunofluorescence was used to analyze the distribution of exosomes. As shown in Figure 6D, more PKH26-positive SBC-3 cells were observed, indicating that SBC-3 cells could effectively uptake these exosomes.

We measured miR-92b-3p and PTEN expression in SCLC cells after co-culturing with SCLC OE cell-derived exosomes and found that cells showed higher miR-92b-3p levels and lower PTEN levels (Figure 6E). We further assessed the effect of exosomes on cell migration. As expected, SBC-3 cells displayed high migration ability after incubation with SBC-3 OE cell exosomes (Figure 6F). To explore the role of the exosomes *in vivo*, we established a xenograft model. As shown in Figure 6G, DDP treatment inhibited tumor growth, whereas combination therapy with exosomes significantly reversed the antitumor effect of DDP. Taken together, these findings demonstrated that miR-92b-3p could be secreted by SCLC OE cells and then delivered via exosomes to promote cell migration and chemoresistance by mediating PTEN expression.

### Plasma miR-92b-3p Expression Is Associated With Chemoresistance and Prognosis in Small Cell Lung Cancer Patients

**FIGURE 7:**
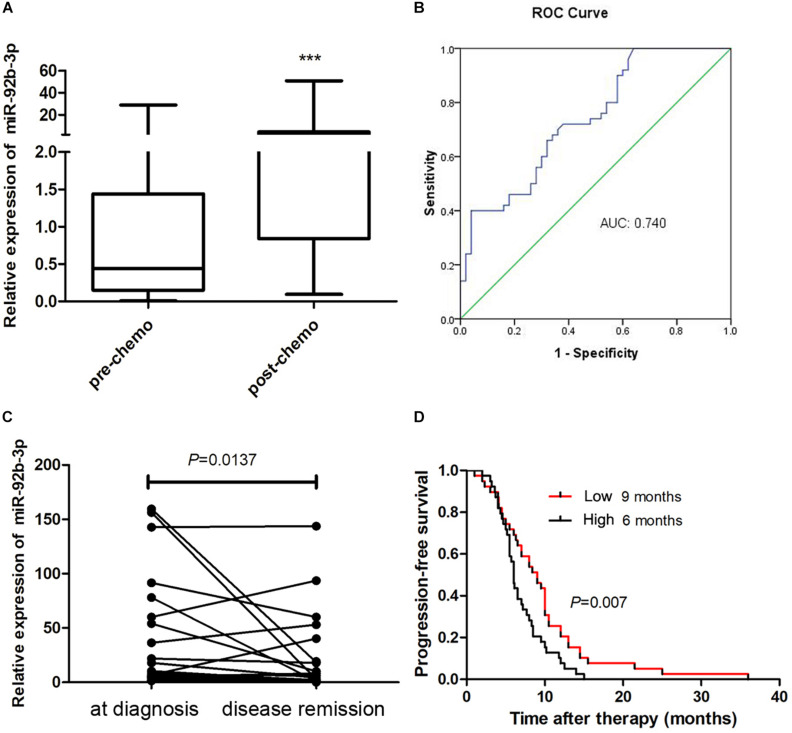
MiR-92b-3p is a biomarker for monitoring the chemoresistance and prognosis of SCLC patients. **(A)** The expression of plasma miR-92b-3p in pre- and post-chemoresistant SCLC patients. **(B)** ROC curve analysis of plasma miR-92b-3p in pre- and post-chemoresistant SCLC patients. ***P < 0.001. **(C)** The level of circulating miR-92b-3p was particularly high in the plasma samples of SCLC patients who achieved disease remission. **(D)** Kaplan–Meier analysis showed that miR-92b-3p is a risk factor for the prognosis of SCLC patients. SCLC, small cell lung cancer and ROC, receiver operating characteristic.

To better understand the clinical value of miR-92b-3p in patients with SCLC, we measured the miR-92b-3p level in plasma samples. We found that plasma miR-92b-3p levels were significantly higher in patients with chemoresistant SCLC than in patients with chemosensitive SCLC (Figure 7A). Receiver operating characteristic (ROC) curves were plotted to determine the diagnostic efficacy of plasma miR-92b-3p for monitoring chemoresistance in 50 SCLC patients. The area under the ROC curve (AUC) of miR-92b-3p was 0.74 (Figure 7B), indicating that miR-92b-3p was a potential biomarker that could distinguish pre- and post-chemoresistant SCLC patients. We next investigated whether miR-92b-3p could be used as a biomarker for monitoring the chemotherapy response in 25 SCLC patients. The relative levels of miR-92b-3p were significantly lower in the disease remission group than in the initial diagnosis group (Figure 7C), indicating that it has potential value for evaluating the chemotherapy response. We further conducted a prognostic Kaplan–Meier analysis of 78 SCLC patients. The progression-free survival (PFS) of SCLC patients with high miR-92b-3p expression was significantly shorter than that of patients with low miR-92b-3p expression (6 vs 9 months, *P* = 0.007, Figure 7D), indicating that miR-92b-3p was a risk factor for the prognosis of SCLC patients.

## Discussion

Multidrug resistance is an essential factor contributing to the high morbidity and mortality in SCLC. Therefore, it is important to explore possible targets to prevent the occurrence of chemoresistance (Huang et al., 2019; Peng et al., 2020). In recent years, numerous studies have shown that miRNAs play a key role in regulating the chemoresistance of cancer cells (Dong et al., 2019; Farhan et al., 2019; Yu et al., 2019). Both oncogenic and tumor-suppressive roles of different miRNAs have been reported in various cancers (Gong et al., 2018; Li C. et al., 2019; Wang et al., 2019). It is well recognized that several miRNAs, including miR-495 (Ye et al., 2017), miR-335 (Tang et al., 2016), miR-30a-5p (Yang et al., 2017), and miR-7 (Liu et al., 2015), are involved in SCLC chemoresistance. In the present study, we first analyzed the distribution of different miRNAs in plasma exosomes from SCLC patients at the pre- and post-chemoresistant stages. The data showed that miR-92b-3p levels were significantly increased in patients with chemoresistant SCLC. Similarly, miR-92b-3p expression was enhanced in chemoresistant SCLC cell lines. As expected, miR-92b-3p overexpression reversed the inhibitory effect of chemotherapy drugs on cell proliferation, migration, and invasion and reduced the rate of apoptosis. Moreover, miR-92b-3p overexpression promoted tumor growth in animal experiments. These results suggested that miR-92b-3p accelerated SCLC development and chemoresistance. However, the molecular mechanisms through which miR-92b-3p exerts these functions are still poorly understood.

MicroRNAs binding to the 3′-UTR of target mRNAs usually resulted in translational suppression or mRNA degradation of numerous target genes (Zhang et al., 2014). To gain insight into the molecular mechanisms through which miR-92b-3p regulates SCLC chemoresistance, bioinformatics was used to predict its possible target genes. As shown in the database, PTEN was found to be a target of miR-92b-3p. The PTEN/AKT pathway has been shown as an important target in remodeling of the nervous system. On the one hand, PTEN suppression reactivates the PI3K/AKT/mammalian target of rapamycin pathway, which leads to further neuron growth and protein synthesis (Goschzik et al., 2014). AKT phosphorylation can also suppress the activity of glycogen synthase kinase 3β and further modify microtubule or actin assembly within the axon, thereby contributing to axon regeneration (Liu et al., 2012; Berry et al., 2016). On the other hand, Godena and Ning (2017) demonstrated that PTEN downregulation could inhibit neuronal apoptosis through the AKT pathway. In addition, several studies have shown that the PTEN/PI3K/AKT pathway is involved in chemoresistance in various cancers. Zhao et al. revealed that miR-3142 regulated cell proliferation and chemoresistance through activating the PTEN/AKT pathway in CML (Zhao et al., 2017). Yu et al. (2008) demonstrated that the PI3K/AKT pathway played an important role in the chemoresistance of gastric cancer cells to etoposide and doxorubicin. Yang et al. (2020) reported that miR-1269b promoted DDP resistance in human NSCLC by modulating the PTEN/PI3K/AKT signaling pathway. Consistent with these findings, we predict that the PTEN/AKT pathway is involved in regulating SCLC chemoresistance. In our study, luciferase activity analysis confirmed that miR-92b-3p could directly target PTEN. We further observed that miR-92b-3p exerted the regulatory function by decreasing PTEN levels and consequently promoted phosphorylation of its downstream target, AKT, *in vitro* and *in vivo*. Additionally, p-AKT downregulation observably reduced cell migration and chemoresistance and promoted PTEN but reduced miR-92b-3p expression in SCLC cells. These data indicated that miR-92b-3p promoted SCLC chemoresistance through the PTEN/AKT pathway.

As secreted miRNAs, exosomal miRNAs appear to be appropriate as ideal biomarkers of cancers due to their non-invasive features. Tumor-derived exosomes contain multiple miRNAs involved in carcinogenesis, cell migration, invasion, and chemoresistance in various cancers (He et al., 2019). One probable explanation for these inconsistent effects may relate to the complex interactions among intercellular environmental factors, exosomes and recipient cells. Several serum exosomal miRNAs serve as predictive markers for chemoresistance in advanced colorectal cancer (Jin et al., 2019). Exosomes derived from gemcitabine-resistant cells can confer malignant phenotypes to target cells by delivering miRNA-222-3p (Wei et al., 2017). Li D. et al. (2020) reported that exosomal miR-613 could reverse resistance to DDP in NSCLC. Ma et al. (2019) showed that exosomes could transfer DDP-induced miR-425-3p to confer chemoresistance in NSCLC. However, few studies have identified the role of exosomal miRNAs in SCLC. In the present study, exosomes were extracted from the conditioned medium of SCLC cells overexpressing miR-92b-3p. Our results showed that these exosomes significantly promoted SBC-3 cell migration and chemoresistance *in vitro* and observably reduced the inhibitory effect of DDP on tumor growth *in vivo*. Moreover, SCLC cells incubated with exosomes showed lower PTEN levels and higher miR-92b-3p levels, indicating that miR-92b-3p could be transferred via exosomes and then target PTEN to confer chemoresistance in SCLC.

Since miRNAs are highly stable in plasma/serum, they have great potential as the biomarkers in cancer screening and monitoring (Mitchell et al., 2008). Serum levels of miR-21 and miR-92 have been reported to predict recurrence in colon cancer patients (Conev et al., 2015). Plasma miR-92a-2 is a potential biomarker for SCLC diagnosis (Yu et al., 2017). In our study, we found that plasma miR-92b-3p levels were significantly increased in chemoresistant SCLC patients, and the AUC was 0.74. Conversely, plasma miR-92b-3p expression was decreased in patients who achieved disease remission. Moreover, SCLC patients with high miR-92b-3p expression had shorter PFS. These data indicated that the plasma miR-92b-3p level has potential value for monitoring the chemoresistance, chemotherapy response, and prognosis of SCLC patients.

Certainly, our study possessed some limitations. First, our previous findings showed above indicated that circulating miR-92b and miR-375 might be ideal non-invasive biomarkers for monitoring the drug resistance during chemotherapy and evaluating the prognosis of the patients with SCLC (Li M. et al., 2020). The function and underlying molecular mechanism involved in miR-375-regulated the chemoresistance of SCLC remain unclear. Second, there are other potential target pathways for the miR-92b-3p associated with SCLC chemoresistance. Thus, more studies are needed to the unresolved areas in the future.

## Conclusion

In summary, we investigated the involvement of the PTEN/AKT regulatory network in miR-92b-3p-mediated cell migration and chemoresistance *in vitro* and *in vivo*. In addition, our study verified that exosomes could transfer miR-92b-3p to promote the development of SCLC chemoresistance. Moreover, circulating miR-92b-3p might be a potential dynamic biomarker to monitor the chemoresistance, chemotherapy response, and prognosis of SCLC patients. Our data will provide new insights for the SCLC treatment and also lay a foundation for the clinical application of exosomal miRNAs.

## Data Availability Statement

The datasets presented in this study can be found in online repositories. The names of the repository/repositories and accession number(s) can be found below: Gene Expression Omnibus (GEO) database under accession number GSE168436.

## Ethics Statement

The studies involving human participants were reviewed and approved by The Institutional Animal Care and Use Committee of The First Affiliated Hospital of University of Science and Technology of China. The patients/participants provided their written informed consent to participate in this study. The animal study was reviewed and approved by The Institutional Animal Care and Use Committee of The First Affiliated Hospital of University of Science and Technology of China.

## Author Contributions

WL and ML designed the project. YH, FC, YC, WS, LL, and XD collected samples and performed the clinical study. WS and ML designed the analysis and analyzed the data. WS wrote the manuscript. WL, ML, WS, LL, XB, JZ, and HL revised the manuscript. All authors contributed to the article and approved the submitted version.

## Conflict of Interest

The authors declare that the research was conducted in the absence of any commercial or financial relationships that could be construed as a potential conflict of interest.
